# Therapeutic Challenges and Emerging Strategies for *T674I* and *PTPN11* Mutations in a *FIP1L1-PDGFRA*-Positive Myeloproliferative Neoplasm: A Case Report

**DOI:** 10.3390/life15030505

**Published:** 2025-03-20

**Authors:** Sıdıka Gülkan Özkan, Ali Kimiaei, Seyedehtina Safaei, Mutlu Karkucak, Mustafa Nuri Yenerel, Aslı Yüksel Öztürkmen, Burak Alp, Hasan Atilla Özkan

**Affiliations:** 1 Department of Internal Medicine, Division of Hematology, Faculty of Medicine, Bahçeşehir University, 34734 Istanbul, Turkey; drgulkan@gmail.com (S.G.Ö.); tinasafaei@outlook.com (S.S.); atillaozkan@yahoo.com (H.A.Ö.); 2 Genetic Diseases Assessment Center, İstinye University, 34010 Istanbul, Turkey; mutlukarkucak@hotmail.com; 3 Department of Internal Medicine, Division of Hematology, Istanbul Medical Faculty, Istanbul University, 34093 Istanbul, Turkey; mnyenerel@gmail.com; 4 Department of Internal Medicine, Division of Hematology, Sivas Numune Hospital, 58060 Sivas, Turkey; yagkbln@gmail.com; 5 Adult Hematology and Bone Marrow Transplantation Unit, Medical Park Göztepe Hospital, 34732 Istanbul, Turkey; burakalp316@gmail.com

**Keywords:** eosinophilic disorder, *FIP1L1-PDGFRA T674I* mutation, imatinib resistance, *PTPN11* mutation, myeloproliferative neoplasm

## Abstract

Myeloproliferative neoplasm (MPN) with eosinophilia associated with *FIP1L1-PDGFRA* is a rare eosinophilic disorder typically treated with imatinib. However, resistance due to the T674I mutation poses a significant challenge. This case presents the first reported instance of concurrent *FIP1L1-PDGFRA T674I* and *PTPN11* (p.E76D) mutations in a 38-year-old male patient with MPN and eosinophilia. The patient initially responded to imatinib but developed resistance after ten months, leading to severe spinal cord compression caused by granulocytic sarcoma. Despite undergoing radiotherapy, chemotherapy, and allogeneic hematopoietic stem cell transplantation (allo-HSCT), the disease progressed. Although full donor chimerism was achieved post-transplant, the patient relapsed shortly afterward with eosinophilia, splenomegaly, and constitutional symptoms. Further treatments, including sorafenib and decitabine, failed to control the disease, and the patient ultimately died from multiorgan failure. This case illustrates the therapeutic challenges associated with *FIP1L1-PDGFRA T674I*-positive eosinophilic disorder, especially when compounded by the *PTPN11* mutation. Resistance to standard treatments underscores the urgent need for novel therapies to manage this rare and aggressive disease.

## 1. Introduction

Hypereosinophilic syndrome (HES) is a rare hematologic disorder characterized by the chronic overproduction of eosinophils in the bone marrow, leading to elevated eosinophil counts in the bloodstream [1]. The criteria used to define HES are as follows: (1) blood eosinophilia of ≥1500/mm^3^ lasting for more than 6 months (or death before 6 months with signs and symptoms suggestive of hypereosinophilic disease), (2) absence of evidence indicating parasitic, allergic, or other known causes of eosinophilia, and (3) probable signs of organ involvement, including heart failure, gastrointestinal issues, central nervous system abnormalities, fever, or weight loss [2,3].

If secondary causes of eosinophilia are excluded, the next step is to assess for a primary bone marrow disorder [4]. This involves analyzing the blood smear and conducting tests for circulating blasts, dysplastic cells, monocytosis, and elevated levels of serum B12 or tryptase [4]. These findings, combined with bone marrow analysis through morphological, cytogenetic, and immunophenotypic evaluation, can assist in determining whether the differential diagnosis of eosinophilia includes a clearly defined myeloid neoplasm according to the World Health Organization [4].

Myeloproliferative neoplasm (MPN) with eosinophilia associated with *FIP1L1-PDGFRA* is a form of hypereosinophilic syndrome [4]. It falls under the category of myeloid/lymphoid neoplasms with eosinophilia and rearrangements involving *PDGFRA*, *PDGFRB, FGFR1*, or *PCM1-JAK2*, as classified in the 2016 World Health Organization classification of myeloid neoplasms [4].

All HESs are expected to have an annual age-adjusted incidence rate of 0.18 to 0.36 per 100,000 person-years [1]. Only a small percentage of affected individuals have genetic abnormalities, with the most prevalent being fusion involving *FIP1L1-PDGFRA*. According to recent data, the average yearly incidence of *FIP1L1-PDGFRA*-positive myeloid neoplasm with eosinophilia is 0.18 cases per one million people, with a predominance in males [5].

**Figure 1:**
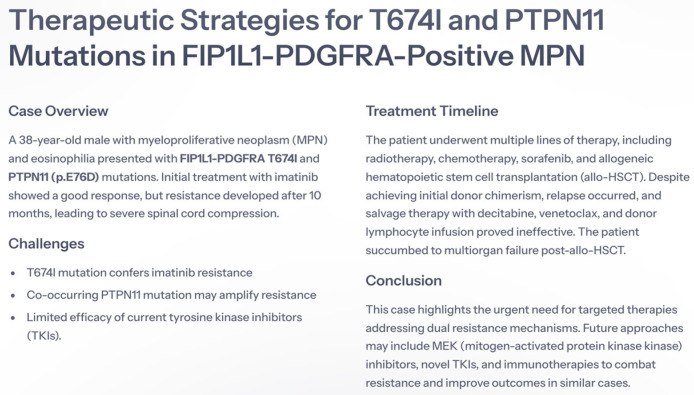
An overview of therapeutic challenges and treatment strategies for a patient with *FIP1L1-PDGFRA T674I* and *PTPN11*-mutated myeloproliferative neoplasm. The figure summarizes the patient’s disease progression, resistance to tyrosine kinase inhibitors, treatment responses, and post-transplant outcomes.

Imatinib mesylate, a tyrosine kinase inhibitor, is the first-line treatment for individuals with the *FIP1L1-PDGFRA* mutation [6]. The therapeutic effects have been demonstrated with a low dose of 100–200 mg per day, which resulted in an excellent response, while a lower dose of 100–200 mg per week effectively maintained remission [7]. Although rare, resistance to imatinib poses significant treatment challenges and has been predominantly linked to the *T674I* mutation [7]. In this report, we present a case of a patient with *FIP1L1-PDGFRA T674I*-positive MPN with eosinophilia, harboring a *PTPN11* (p.E76D) mutation. Figure 1 illustrates the therapeutic timeline, genetic mutations, and treatment challenges encountered in this patient.

## 2. Detailed Case Description

A 38-year-old male patient presented with weakness, bone pain, and B symptoms, prompting him to seek medical attention. He had no known history of chronic illnesses. Physical examination was normal, without massive splenomegaly. Bone marrow examination revealed marked eosinophilia without excess blasts. The patient was diagnosed with *FIP1L1-PDGFRA*-positive myeloid with eosinophilia. A PET CT performed at diagnosis showed no additional findings of advanced splenomegaly.

After initiation of Imatinib 100 mg/day, symptoms were controlled, and a hematologic response was achieved. Molecular testing was not performed during treatment. Ten months after starting Imatinib, severe bone pain began, followed by sudden weakness in the lower extremities. Spinal MRI revealed a 9 cm mass severely compressing the spinal cord at the C7-T3 vertebrae. Decompression surgery and mass biopsy were performed, with the vertebral mass biopsy reported as granulocytic sarcoma.

A PET CT performed 10 months after the first presentation due to the newly occurring vertebral mass showed supraclavicular and cervical lymph nodes with lower FDG uptake than the liver, right pleural effusion (2.5 cm diameter, SUVmax: 4.2), newly developed mediastinal lymph nodes (SUVmax: 3.1), a hypermetabolic area in the rectum (SUVmax: 7.9), a newly developed C7-D2 vertebral mass (SUVmax: 9.2), and a newly developed vertebral mass in D7 (SUVmax: 4.5). Additionally, multiple hypermetabolic foci were observed on the left humerus head, ribs, and vertebrae. A bone marrow biopsy showed eosinophilic involvement without excess blasts.

Radiotherapy was applied to the involved vertebral column area. Imatinib 100 mg/day was continued, and a 3 + 7 chemotherapy regimen was started after radiotherapy 11 months after the first presentation. Thirteen months after the first presentation, a high-dose ARA-C regimen was initiated. MRI scan showed no significant regression in the mass size after treatment, and B symptoms recurred. The patient was referred to our institution for allogeneic hematopoietic stem cell transplantation (allo-HSCT) due to refractory disease.

On initial physical examination, the patient was conscious and cooperative, with a fever of 38.1 °C. Lung and heart auscultation were normal, with mild tachycardia. Ten-centimeter splenomegaly was noted. Muscle strength of the lower extremities was 2/5.

Initial laboratory values prior to hospitalization were as follows: WBC 37,000/μL, RBC 2.9 million/μL, Hb 8.3 g/dL, MCV 85 fL, RDW 18%, platelets 26,000/μL, neutrophil count 18,600/μL, lymphocyte count 1500/μL, and eosinophil count 16,400/μL. A bone marrow biopsy showed an increase in eosinophilic series, maturation arrest at the myelocyte and metamyelocyte stages, a marked decrease in erythroid series and megakaryocytes, a 1–2/3 reticulin fiber increase, and 5% myeloblasts.

**Figure 2:**
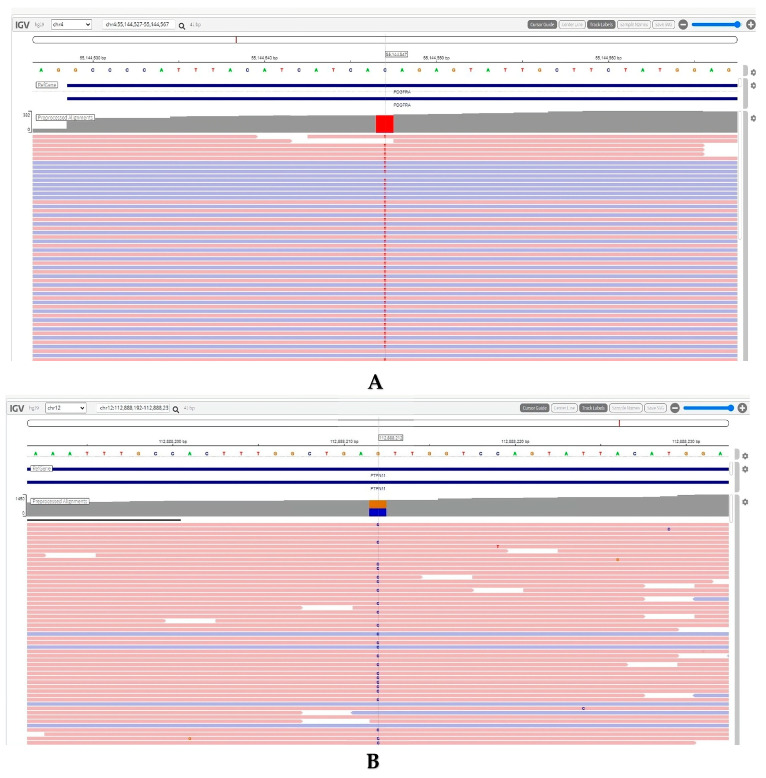
IGV images from the NGS panel results showing mutations in (**A**) the PDGFRA gene (p.T674I) and (**B**) the *PTPN11* gene (p.E76D). Abbreviations: IGV, Integrative Genomics Viewer; NGS, Next-Generation Sequencing; PDGFRA, Platelet-Derived Growth Factor Receptor Alpha; *PTPN11*, Protein Tyrosine Phosphatase Non-Receptor Type 11.

A PET-CT was performed and compared with the pretreatment PET-CT, showing a significant response in lymph nodes and pleural fluid, a complete response in vertebral masses, and a partial response in bone lesions. The *FIP1L1-PDGFRA* test was positive by FISH analysis. Next-generation sequencing was performed using the Archer FusionPlex Myeloid Panel (originally developed by ArcherDX, headquartered in Boulder, Colorado, USA. In December 2022, ArcherDX was acquired by Integrated DNA Technologies (IDT), based in Coralville, Iowa, USA) (RNA extraction with kit ReliaPrep FFPE (Promega Corporation, Madison, WI, USA) total RNA-Promega, RNA amplification and sequencing with Illumina-Novaseq 6000 (Illumina, Inc., San Diego, CA, USA), data analysis with Archer^®^ Analysis Version: 7.3.2 software) and revealed genomic alterations in the *PDGFRA* gene (NM_006206.5) at position c.2021C > T (p.Thr674Ile) and in the *PTPN11* gene (NM_002834.4) at position c.228G > C (p.Glu76Asp) (Figure 2).

The FLAG-IDA treatment regimen was initiated with sorafenib (off-label use). All symptoms resolved with treatment. However, bone pain, B symptoms, and eosinophilia recurred with hematologic recovery.

Due to refractory disease, after the MEL/FLU/TBI (Melphalan 140 mg/m^2^ on day −11, Fludarabine 100 mg/m^2^ between days −5 and −2, TBI 8 Gy between days −3 and −2) conditioning regimen, allogeneic hematopoietic stem cells were transplanted from an HLA-matched, blood group-compatible sister 16 months after the first presentation. Post-transplant cyclophosphamide and cyclosporine were used for graft-versus-host disease prophylaxis. Neutrophil and platelet engraftment occurred on days +14 and +15, respectively. During the neutropenic period, the patient experienced febrile neutropenia and grade 2–3 mucositis.

Chimerism at day +28 showed 100% donor cells in the peripheral blood. B symptoms resolved, and organomegaly was not found. Sorafenib 400 mg twice daily was continued as a maintenance treatment.

Chimerism at day +56 showed 53% donor cells in peripheral blood. A few days later, B symptoms, splenomegaly, and eosinophilia recurred. On day 60 post-allo-HSCT, Decitabine 20 mg/m^2^ for 5 days and Venetoclax 400 mg/day for 14 days were initiated. Sorafenib was discontinued during this period. On the 15th day of treatment, the patient developed jaundice, pleural, and pericardial effusions, and pericardiocentesis was performed due to cardiac tamponade. Pericardial fluid analysis revealed leukocytosis with increased eosinophils. Donor lymphocyte infusion was administered for the refractory disease. Despite these interventions, on day +85 post-allo-HSCT, the patient died due to multiorgan failure and refractory disease.

## 3. Discussion

This case highlights the formidable therapeutic challenges of *FIP1L1-PDGFRA T674I*-positive MPN with eosinophilia with concurrent *PTPN11* (p.E76D) mutation. Despite aggressive multimodal treatment including imatinib, sorafenib, chemotherapy, radiotherapy, and allo-HSCT, the patient experienced rapid disease progression and a poor outcome. The coexistence of *T674I PDGFRA* and *PTPN11* mutations likely drove the aggressive clinical course and therapeutic resistance. No standardized treatment protocol exists for *FIP1L1-PDGFRA*-positive MPN harboring the *T674I* mutation, particularly with additional genetic alterations. This case emphasizes the urgent need for novel therapeutic approaches targeting complex resistance mechanisms in this rare and challenging patient population.

To our knowledge, this is the first reported case of *FIP1L1-PDGFRA*-positive MPN with concurrent *T674I* and *PTPN11* (p.E76D) mutations. While the role of *PTPN11* mutations in *FIP1L1-PDGFRA*-positive MPN has not been previously described, it is considered that this mutation may have contributed to the resistance mechanism observed in this patient.

*PTPN11* encodes SHP-2, a protein tyrosine phosphatase that plays a critical role in the *RAS/MAPK* signaling pathway, which regulates cell proliferation, differentiation, and survival [8]. Germline mutations in the *PTPN11* gene account for approximately 40% to 50% of Noonan syndrome (NS) cases, a prevalent autosomal dominant disorder marked by facial features, proportionate short stature, and heart defects [9]. Individuals with NS often show signs of myeloproliferative disorders (MPD), typically presenting as transient leukocytosis (elevated white blood cell count) and splenomegaly (enlarged spleen), which typically resolve without long-term effects [9]. In rare instances, however, these patients may develop juvenile myelomonocytic leukemia (JMML) [9].

Somatic gain-of-function mutations in this gene, such as *p.Glu76Asp*, lead to hyperactivation of this pathway and have been implicated in various hematological malignancies, including JMML, acute myeloid leukemia (AML), B-cell acute lymphoblastic leukemia (B-ALL), and myelodysplastic syndromes (MDS) [8]. In this case, the *PTPN11* (*p.Glu76Asp*) mutation may have contributed to the aggressive disease course and treatment resistance by promoting cell proliferation, survival, and resistance to apoptosis.

In eosinophilic disorders, for M/LN-eo linked to *ETV6::ABL1*, although available data are limited, mutations in genes such as *ARID2*, *TP53*, *SETD2*, *CDKN1B*, *PTPN11*, and *SMC1A* have been observed in roughly 50% of cases [10]. Likewise, in M/LN-eo with *FLT3* fusions, mutations in genes including *ASXL1*, *PTPN11*, *RUNX1*, *SETBP1*, *SRSF2*, *STAT5B*, *TET2*, *TP53*, and *U2AF1* have been found in approximately 40% to 50% of cases [10].

In the context of *FIP1L1-PDGFRA*-positive MPN with eosinophilia, the concurrent *PTPN11* mutation could have amplified downstream signaling, making the leukemic cells less dependent on *PDGFRA*-driven pathways and more resistant to tyrosine kinase inhibitors like imatinib. This dual activation of signaling pathways (*PDGFRA* and *RAS/MAPK*) may explain the rapid development of resistance and the poor response to subsequent therapies, including sorafenib and decitabine. Furthermore, *PTPN11* mutations are known to confer resistance to chemotherapy and targeted therapies in other cancers, which could account for the failure of high-dose ARA-C in this case [11]. Targeting the *RAS/MAPK* pathway (e.g., with MEK inhibitors) in combination with *PDGFRA* inhibitors could be a potential strategy to overcome resistance in similar cases, although this remains speculative and requires further investigation [8]. While both *PTPN11* mutations and *PDGFRA* rearrangements independently contribute to hematologic malignancies, there is limited evidence to suggest a direct interaction between these genetic alterations in the pathogenesis of such diseases. Further research is needed to elucidate any potential cooperative roles between *PTPN11* mutations and *PDGFRA* activation in hematologic malignancies. This case is significant as it represents a genetic association that has been described for the first time in the literature.

Treatment options for *FIP1L1-PDGFRA*-positive MPN with eosinophilia with *T674I* mutation remain challenging. While imatinib is highly effective for *FIP1L1-PDGFRA*-positive MPN with eosinophilia, the *T674I* mutation confers resistance, necessitating alternative approaches. For instance, nilotinib failed to elicit a response in one patient, prompting a switch to sorafenib [12]. Sorafenib has shown in vitro activity against *T674I* mutants, but clinical responses are often transient due to the emergence of additional resistant mutations like *D842V* [12,13,14]. Ponatinib, a third-generation tyrosine kinase inhibitor, has demonstrated in vitro efficacy against both *T674I* and *D842V* mutations [15]. However, compound mutations can develop, leading to disease progression even under ponatinib treatment [15]. Crenolanib has shown promise in preclinical studies, particularly against *T674I* mutants, but clinical efficacy remains to be established [15].

The rapid development of resistance to various tyrosine kinase inhibitors highlights the need for novel therapeutic strategies. New biologic treatments, including mepolizumab, reslizumab, and benralizumab, have shown effectiveness in managing hypereosinophilic syndrome (HES), especially in severe or treatment-resistant cases [16]. Mepolizumab and reslizumab reduce eosinophil levels, while benralizumab induces eosinophil death through antibody-dependent cell-mediated cytotoxicity [16]. Mepolizumab is FDA-approved for eosinophilic granulomatosis with polyangiitis (EGPA) and can reduce the need for steroids, although its benefits in steroid-resistant HES are limited [17]. Benralizumab is approved for eosinophilic asthma, and its role in HES is still being investigated [18]. Other promising treatments, such as dexpramipexole, AK002/lirentelimab, dupilumab, and JAK2 inhibitors, are also under investigation for HES management [16].

In cases of refractory disease or blast transformation, -HSCT may be considered as a potentially curative option [19]. A retrospective study conducted by the Chronic Malignancies Working Party of the European Society for Blood and Marrow Transplantation (EBMT) revealed that the preferences for conditioning regimens and T cell depletion strategies were heterogeneous [20]. The reported cohort underwent allo-HSCT over an extended period of time [20]. However, outcomes remain poor.

## 4. Conclusions

This case highlights the complexities involved in treating *FIP1L1-PDGFRA T674I*-positive eosinophilic disorder, especially when compounded by additional mutations like *PTPN11*. Despite initial responses to imatinib and allo-HSCT, the rapid emergence of resistance emphasizes the limitations of current treatment options. These complex resistance patterns call for the development of new targeted therapies and combination strategies to enhance outcomes in such challenging cases. The poor prognosis associated with refractory cases involving multiple mutations further underscores the urgent need for innovative targeted treatments and combination approaches to improve outcomes in this rare and difficult-to-treat condition.

## Data Availability

The data supporting the findings of this study are available from the corresponding author upon reasonable request. Due to patient confidentiality, some data may be restricted.

## Abbreviations

ARA-C	Cytarabine
allo-HSCT	Allogeneic Hematopoietic Stem Cell Transplantation
B symptoms	Fever, night sweats, and weight loss
M/LN-eo	Myeloid and lymphoid neoplasms with eosinophilia
MPN	Myeloproliferative neoplasm
CT	Computed Tomography
FDG	Fluorodeoxyglucose
*FIP1L1-PDGFRA*	Fusion of FIP1-Like 1 and Platelet-Derived Growth Factor Receptor Alpha
FLAG-IDA	Fludarabine, Cytarabine, and Idarubicin regimen
FISH	Fluorescence In Situ Hybridization
FLU	Fludarabine
GVHD	Graft-Versus-Host Disease
Hb	Hemoglobin
HES	Hypereosinophilic Syndrome
HLA	Human Leukocyte Antigen
MCV	Mean Corpuscular Volume
MEL	Melphalan
MRI	Magnetic Resonance Imaging
PET	Positron Emission Tomography
*PTPN11*	Protein Tyrosine Phosphatase Non-Receptor Type 11
RBC	Red Blood Cell
RDW	Red Cell Distribution Width
SUVmax	Maximum Standardized Uptake Value
TBI	Total Body Irradiation
TKI	Tyrosine Kinase Inhibitor
WBC	White Blood Cell
